# Demonstrating the In Vitro and In Situ Antimicrobial Activity of Oxide Mineral Microspheres: An Innovative Technology to Be Incorporated into Porous and Nonporous Materials

**DOI:** 10.3390/pharmaceutics15041261

**Published:** 2023-04-17

**Authors:** Katia Iskandar, Sophie Pecastaings, Céline LeGac, Sylvie Salvatico, Catherine Feuillolay, Mylène Guittard, Loïc Marchin, Marc Verelst, Christine Roques

**Affiliations:** 1 Department of Pharmacy, School of Pharmacy, Lebanese International University, Bekaa P.O. Box 146404, Lebanon; 2 National Institute of Public Health, Clinical Epidemiology, and Toxicology-Lebanon (INSPECT-LB), Beirut 6573, Lebanon; 3 Laboratoire de Génie Chimique, Faculté de Pharmacie, Université de Toulouse, CNRS, INPT, UPS, 31062 Toulouse, France; sophiepecataings@airliquide.com (S.P.); christine.roques@univ-tlse3.fr (C.R.); 4 FONDEREPHAR, Faculté de Pharmacie, 31062 Toulouse, France; celine.legac@fonderephar.com (C.L.); sylvie.salvatico@fonderephar.com (S.S.); catherine.feuillolay@fonderephar.com (C.F.); 5 Pylote SAS, 22 Avenue de la Mouyssaguèse, 31280 Drémil-Lafage, France; mguittard@pylote.com (M.G.); loic.marchin@pylote.com (L.M.); 6 CEMES, UPR CNRS 8011, 29 Rue Jeanne Marvig, CEDEX, 31055 Toulouse, France; marc.verelst@univ-tlse3.fr

**Keywords:** oxide mineral microspheres, antimicrobial surface, simulation of use conditions, in situ

## Abstract

The antimicrobial activity of surfaces treated with zinc and/or magnesium mineral oxide microspheres is a patented technology that has been demonstrated in vitro against bacteria and viruses. This study aims to evaluate the efficiency and sustainability of the technology in vitro, under simulation-of-use conditions, and in situ. The tests were undertaken in vitro according to the ISO 22196:2011, ISO 20473:2013, and NF S90-700:2019 standards with adapted parameters. Simulation-of-use tests evaluated the robustness of the activity under worst-case scenarios. The in situ tests were conducted on high-touch surfaces. The in vitro results show efficient antimicrobial activity against referenced strains with a log reduction of >2. The sustainability of this effect was time-dependent and detected at lower temperatures (20 ± 2.5 °C) and humidity (46%) conditions for variable inoculum concentrations and contact times. The simulation of use proved the microsphere’s efficiency under harsh mechanical and chemical tests. The in situ studies showed a higher than 90% reduction in CFU/25 cm^2^ per treated surface versus the untreated surfaces, reaching a targeted value of <50 CFU/cm^2^. Mineral oxide microspheres can be incorporated into unlimited surface types, including medical devices, to efficiently and sustainably prevent microbial contamination.

## 1. Introduction

Recent advances in antimicrobial material technologies are revolutionizing infectious disease management by inhibiting biofouling, contamination, and infection [1,2,3]. The antimicrobial surface activity is exerted (i) by killing the micro-organisms and/or (ii) by preventing their adhesion to inanimate surfaces [1,4], leading to the inhibition of microbial survival, growth, and potential biofilm formation [1,4]. Available antimicrobial surfaces rely on the modulation of surface topography, wettability, and chemistry [4]. Conventional surfaces can be (1) biocides for contact-killing [5] and (2) biocides for release-killing [6,7].

The biocides for release-killing rely on the biocidal effect of metals such as silver (Ag), copper (Cu), gold (Au), titanium (Ti), and zinc (Zn) [6,7]. The nanocomposites and nanoparticles (NPs) of these metal oxides, such as CuO, Fe_3_O_4_, ZnO, MgO, and TiO_2_, demonstrated better antimicrobial properties than the parent metal’s NPs [7,8,9,10,11,12,13]. They primarily act by damaging the bacterial cell wall through electrostatic interaction, generating reactive oxygen species (ROS), leading to oxidative stress and disrupting protein functions and bacterial cell structure by releasing metal cations [8,14]. Their potential mutagenicity [8] and their detrimental impact if released into the environment are of concern [15]. A recent meta-analysis highlighted the significant amount of controversy and uncertainty in the published literature related to the antibacterial mechanism of metals and oxide mineral nanomaterials [15] and their contribution to the induction of multidrug-resistant (MDR) bacterial strains [2,16] and biofilm formation [17,18,19,20,21]. Other biocides for release-killing include metal NPs, such as silver nanoparticles (AgNPs) [3,22,23,24], antibiotics [25,26,27], and nitric oxide (NO) [21,28,29].

The biocides for contact-killing [5] include antimicrobial peptides (AMPs), synthetic AMPs mimics, antimicrobial enzymes (AMEs), natural biocidal polymers, and polycations derived from quaternary ammonium compounds (QACs), which are commonly used [5]. Their mechanism of action varies depending on the biocide [5]. The antimicrobial surfaces lead to bacterial cell membrane disruption and oxidative stress [2]. This effect may not be long-lasting [2] and is interrupted once the biocidal agent is depleted [2]. Additional limitations, such as their biocompatibility with the surface [30] and their potential contribution to antimicrobial resistance and toxicity to humans and the environment, are of concern [2]. Bacteria-killing strategies also include photothermal bactericidal surfaces and light-activated killing [14,21,31,32,33,34,35,36]. The bacteria-resisting approach consists of modifying the material’s hydrophilic properties that generate repulsive forces to prevent bacterial adhesion [34,37,38,39] and low-surface-energy materials that repel bacterial adhesion at the initial stage through a mechanical turbulence mechanism [34,40,41,42]. Additional antimicrobial surfaces include patterned surfaces that may originate from natural sources [43,44,45,46,47,48,49,50,51] and functionalized antimicrobial surfaces that are chemically activated using nonleachable materials, and other functional surfaces that are near-infrared (NIR) and UV activated [4,52,53].

The efficiency of antimicrobial surfaces shown in vitro may be of limited applicability in situ [54]. Under normal environmental conditions, the reproducibility and comparability of in vitro and in situ results are difficult to undertake [54,55].

This study presents a unique patented mineral technology manufactured by Pylote SAS, France [56,57]. This breakthrough innovation addresses most of the limitations of antimicrobial surfaces. The technology consists of oxide mineral microspheres (nonrelease, non-ionic, nonmetal, and not nanosphere-based) for green-tech ceramic innovation that is potentially friendly to humans and the environment.

**Figure 1:**
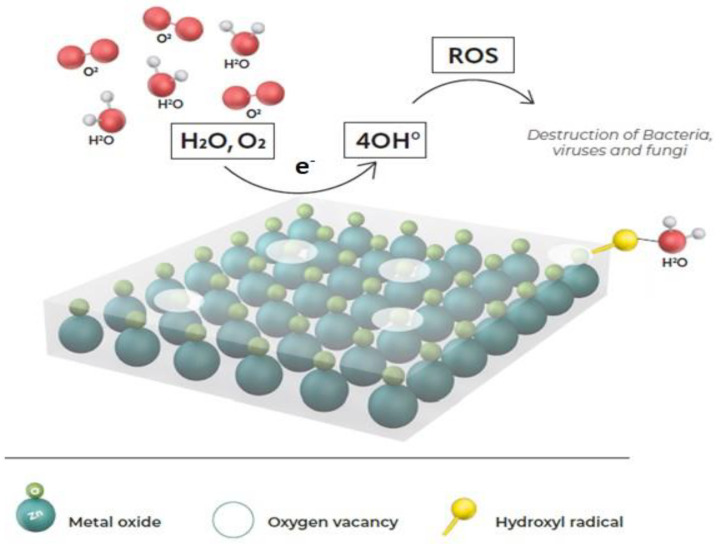
Mechanism of action of oxide mineral microspheres.

The Pylote SAS patented mineral technology is a micromanufactured one-step cleantech process called Pyrolyse pulvérisée^®^ [58]. The microspheres are high-purity ceramic particles with a sphericity coefficient of ≥0.75 µm, characterized by a narrow distribution size. The antimicrobial activity of oxide mineral microspheres is a nonrelease approach exerted upon direct contact with the micro-organism. The oxide mineral microspheres have an electron donor characteristic that produces (when in contact with water) reactive oxygen species (ROS), mainly hydroxyl radicals [59]. The mechanism of action is not photo-activated and relies on the oxide mineral surface defects called oxygen vacancies [60]. The highly oxidizing hydroxyl radicals generated on the surface of the microspheres lead to the destruction of a demonstrated wide array of micro-organisms, including Gram-positive bacteria (GPB), Gram-negative bacteria (GNB), viruses, and at lower levels, fungi by direct contact [58]. This reaction occurs in nanoseconds within dozens of nanometers of the integrated material surface [58] (Figure 1). In addition, the oxidizing agents, including zinc oxide (ZnO) and magnesium oxide (MgO), are rapidly biodegradable active molecules [61,62,63]. The microspheres can be integrated into variable materials during the conventional converting process without any modification on the manufacturing process, impact, or need for additional investment. The surfaces can be porous and nonporous, such as with the containers used in variable sectors, including the pharmaceutical and medical fields. The mineral microspheres are authorized additives for pharmaceutical containers in the European, United States, and Japanese Pharmacopeia [58]. This study aims to demonstrate, according to the ISO 22196:2011, JIS Z 2801, ISO 20743:2021, and NF S90-700:2019 standards, the antimicrobial activity (i) of different types of surfaces with added oxide mineral microspheres in vitro, (ii) the sustainability of this effect under real-life conditions, worst-case scenarios, and (iii) in situ in two different types of premises (ISO 8 room and high school self-service) under real-life use conditions.

## 2. Materials and Methods

### 2.1. In Vitro Assays

Methodologies described in the ISO 22196:2011 (JIS Z2801:2010) [64], ISO 20743:2021 [65], and NF S90-700:2019 [66] standards were applied with some modifications to study the antibacterial activity of microspheres on nonporous and porous surfaces. The tests were conducted three times on a minimum of three samples.

#### 2.1.1. Tested Surfaces

Tested materials and reference materials are described in Table 1 according to the standard applied.

Regarding their treatment before assay:

- For nonporous surfaces, the test pieces (untreated=control=C and treated=assay=A) were prepared by immersion in ethanol 70°, rinsed with distilled sterile water, and then dried under a microbiological safety cabinet before the test according to ISO 22196:2011 and NF S90-700:2019;
- For porous surfaces, the test pieces (untreated=control and treated=assay) were cut in a round shape (diameter: 3 and 8 cm) and sterilized (121 °C, 15 min) according to the ISO 20473:2021 standard.

#### 2.1.2. Bacterial Strains and Growth Conditions

The referenced bacteria (*S. aureus* CIP 4.83 and *E. coli* CIP 53.126 (Institute Pasteur Collection; Paris, France) were tested, along with two bacterial strains expressing acquired mechanisms of resistance: Methicillin-resistant *S. aureus* ATCC 33,591 (MRSA) obtained from the American Type Culture Collection and a clinical isolate of Extended-Spectrum Beta-Lactamase (ESBL)-producing *E. coli* (Institut Fédératif de Biologie (IFB), CHU Toulouse, France). Bacterial strains were preserved at −80 °C.

Before each experiment, a frozen microbial sample was spread on a trypticase-soy agar plate (Biomérieux, Crapone, France) and incubated at 35 °C under aerobiosis for 16 h. A second pre-culture consisted of transferring each strain on a new agar plate incubated at a temperature of 36 °C for 16 h. The second and third precultures serve for tests.

#### 2.1.3. Antimicrobial Surface Activity Testing

Testing conditions followed the ISO 22196:2011, ISO 20473:2013, and NF S90-700:2019 standards at a temperature of 35 ± 1 °C, a relative humidity (RH) > 90%, and a contact time of 24 h with an inoculum size of 10^4^ units per cm^2^. The testing conditions were modified to study the antimicrobial surface activity under conditions close to real uses.

##### Inoculation of the Test Pieces

- For nonporous surfaces, assays referred to two different standards:
  According to ISO 22196:2011 (JIS Z2801:2010), bacterial suspensions were prepared in 1/500 nutrient broth (3.0 g of meat extract, 10.0 g of peptone, and 5.0 g of sodium chloride in 1000 mL of distilled or deionized water) to obtain a concentration able to give rise to a final inoculation ranging between 10^2^ CFU/cm^2^ to 10^5^ CFU/cm^2^. The inoculum was adapted to evaluate the impact on the antimicrobial activity level. A defined volume of the diluted suspension was deposited and spread on the tested surface. For some assays, inoculated pieces were recovered with a sterile polyethylene film (4 cm × 4 cm for a surface of 16 cm^2^) as indicated for hydrophobic surfaces (ISO 22196:2011 and ISO 20473:2013 standards);
  According to FR S90-700, bacterial suspensions were prepared in distilled sterile water and adjusted to 10^6^ CFU/mL. Then, a 1 µL drop was deposited on the surfaces under assay and let under ambient conditions (20 ± 2.5 °C at an RH of 50%) for rapid drying (<3 mn);

- For porous surfaces, assays referred to ISO 20473:2021:
  According to the standard, 1 mL of test suspension prepared in tryptone salt broth (BioMérieux) and adjusted at 106 CFU/mL was deposited on a transfer agar plate (Trypticase Soy Agar-Biomerieux). After 5 min, a cylindric weight (200 g) was applied to the test pieces for 1 min. After contact, test pieces were placed in sterile flasks (transfer surface above) for incubation.

##### Incubation

Tests and controls were placed at 35 ± 1 °C with RH > 90% during the defined contact time (24 h) for assays under ISO 22191:2011 and ISO 20473 standards’ conditions. Other conditions were tested and specified in the results section. For assays undertaken under the FR S90-700 standard, the contact time is 24 h at a temperature of 20 ± 2.5 °C at an RH of 50%.

##### Microbial Recovery from Test Pieces

- For nonporous surfaces, at the end of the contact time, the residual viable micro-organisms were recovered by deposition of 10 mL of Soybean Casein Digest broth with Lecithin and Polyoxyethylene sorbitan monooleate (SCDLP) (17 g casein digest peptone, 3 g Soybean digest peptone, 5 g sodium chloride, 2.5 g potassium hydrogen phosphate, 2.5 g glucose, 1 g lecithin, 7 g polysorbate 80), with sterile glass beads on the tested surface and vortexing the mixture. The obtained suspension was diluted (10-fold dilutions), and 1 mL of the initial suspension and each dilution was poured in Trypticase soy agar (Biomérieux). After incubation at 36 °C for 48 h, colony-forming units (CFU) were enumerated, and the value was expressed as CFU per cm^2^ and then to log CFU per cm^2^. According to the protocol, the detection limit is 10 CFU/surface, i.e., for a surface of 16 cm^2^, 0.63 CFU/cm^2^. CFU corresponds to the number of colonies and, therefore, to a whole number. For this reason, the value of 0.63 has been rounded up to 1. The log 10 of the value 1 equals 0. Thus, an absence of colonies under the test conditions is equivalent to 0 log CFU/cm^2^.
- For porous surfaces, at the end of the contact time, untreated and treated pieces were deposited in sterile flasks with 20 mL of neutralizing solution (Polysorbate 80, Egg yolk lecithin, histidine chloride, casein peptone, mono potassium phosphate, disodium phosphate dihydrate), then mixed for 5 × 5 s with a vortex. The obtained suspension was diluted (10-fold dilutions). A volume of 1 mL from the baseline and each diluted suspension was poured into Trypticase soy agar (Biomérieux). After incubation at 36 °C for 48 h, colony-forming units (CFU) were counted, and the value was expressed as CFU per cm^2^ and then to log CFU per cm^2^.

Tests were repeated three times during the same assay.

Controls (C0 and C24 h) were tested using test pieces without microsphere incorporation (C) to check the lack of antimicrobial activity and to calculate the log reduction.

##### Validation

Validation of each assay followed the indication of the ISO 22196:2011 and ISO 20473:2021 standards, considering that the logarithmic value of the number of viable bacteria recovered immediately after inoculation (T0) from the untreated pieces shall satisfy the following equation:

(Lmax − Lmin)/(Lmean) ≤ 0.2

where

- Lmax is the maximum logarithmic number of viable bacteria;
- Lmin is the minimum logarithmic number of viable bacteria;
- Lmean is the average logarithmic number of viable bacteria of three untreated test pieces.

Validation of each assay followed the indication for NF 90-700 standard, considering that the suspension is in the range 5.18–5.70 log, that the logarithmic value of the number of viable bacteria for the control of dilution-neutralization is not more than 2 log from the deposition with a difference ≤0.3 logs between the two controls of dilution-neutralization and (Lmax − Lmin)/(Lmean) ≤ 0.3 for the three values obtained at T0 for the reference surface during the same assay.

##### Antimicrobial Surface Activity Calculation

The antibacterial activity (R) represents the logarithmic reduction/cm^2^ in the number of bacteria between the Control and Assay surfaces after 24 h of contact according to the following matrix:

R = (C24h − C0) − (A24h − C0) = C24h − A24h

where:

- R is the logarithmic reduction;
- C0 is the average of logarithmic numbers (CFU per cm^2^) of viable micro-organisms recovered from the untreated pieces (controls) immediately after inoculation;
- C24h is the average of logarithmic numbers (CFU per cm^2^) of viable micro-organisms recovered from the untreated test pieces (controls) after 24 h;
- A24h is the average logarithmic numbers (CFU per cm^2^) of viable micro-organisms recovered from the treated test pieces (microsphere-added) after 24 h.

#### 2.1.4. In Vitro Antimicrobial Surface Activity Characterization

Antimicrobial surface activity tests, as previously described, were performed in different assay conditions to characterize the antimicrobial properties of the innovative process of homogeneous incorporation of oxide mineral microspheres into materials.

##### Spectrum of Activity

In the first step, the antimicrobial activity of the microsphere-added (2.5% *w/w*) nonporous surfaces was tested against (1) the two reference bacteria recommended by the ISO 22196:2011 standard (*E. coli* CIP 53.126 and *S. aureus* CIP 4.83) and expanded to antibiotic-resistant bacteria, and (2) against *S. aureus* CIP 4.83 recommended by NF S90-700:2019. The antimicrobial activity of microsphere-added (2.5% *w/w*) porous surfaces was examined against *S. aureus* CIP 4.83, the reference bacteria recommended by the ISO 20743 standard.

##### Nonporous Surfaces: Impact of Contact Time, Relative Humidity, and Temperature

Antimicrobial activity assays were undertaken by varying the contact time (0.5–24 h) between the plastic as well as other nonporous surfaces and *E. coli* CIP 53.126 to determine the minimum time required to obtain a significant effect using different compositions and forms of plastic surfaces (polyethylene (PE), polypropylene (PP), and PP film) containing various concentrations of microspheres (0.5–4% *w/w*).

- The temperature varied from 36 ± 1 °C to 20 ± 2.5 °C and 22 ± 2 °C and the humidity from >90% to between 24% and >80%;
- The contact time varied between 30 min and 1–6 h depending on the tested surface;
- The size of the inoculum ranged between 10^2^ CFU/cm^2^ and 10^5^ CFU/cm^2^;
- The volume of the inoculum was also tested (50 µL versus 400 µL in the standard) to evaluate the drying time impact.

According to the conditions NF S90-700:2019 standard and following the method of ISO 22196:2011, the testing conditions against *S. aureus* CIP 4.83 were changed to 20 ± 2.5 °C at an RH of 50% and a contact time of 24 h.

##### Simulation of Use

A worst-case scenario simulation test undertaken under indicated chemical, physical and mechanical conditions depending on the material is finally used to evaluate the stability of the antimicrobial activity in vitro. The antimicrobial surface activity of the microsphere-added plastic and other nonporous surfaces was checked after 7 and 12 weeks at 50 °C according to the ASTM F 1980-7 standard (*E. coli* CIP 53.126 according to the ISO 22196:2011 standard). The antimicrobial activity was also tested after 100 washing cycles with selected detergents/disinfectants frequently used in hospitals. The detergents/disinfectants were diluted at the recommended doses of use in hospitals: Sodium hypochlorite solution (NaClO) at 0.9%, Primactyl (dodecyl dimethyl ammonium, CAS: 7173-51-5, 2.5 g/kg, ethyl alcohol, CAS: 64-17-5, 125 g/kg—propane-2-ol, CAS: 67-63-0, 104 g/kg.) à 2.4%, isopropyl alcohol, Surfanios Premium (51 mg g^−1^ of N-(3-aminopropyl)-N-dodecylpropane-1,3-diamine and 5 mg g^−1^ of didecyldimethylammonium chloride, Laboratoires Anios, France) diluted at 0025%, and ready-to-use product.

### 2.2. In Situ Assays

Two tests were conducted in public places to examine the CFU/25 cm^2^ of surfaces (high-touch areas) covered versus noncovered with treated polyethylene film. We compared the covered treated surfaces with none to represent the actual in situ characteristics of high-touch areas. (Table 2)

The first test was undertaken in an ISO 8 cleanroom during regular working hours. Sampling was performed according to ISO 144698-2 standard on five high-touch areas, including a bench, door handle, mouse tablet, remote control device, and hydroalcoholic gel button. The microbial burden was examined by taking samples weekly for seven consecutive weeks, every Monday or Tuesday from April to June 2021. Routine cleaning and disinfection procedures were maintained. Count-tact^®^ plates (Biomérieux) were used with the corresponding applicator (500 g precision during 10 s) to obtain samples between 8:00 a.m. and 9:00 a.m. during regular working hours and under normal environmental conditions of temperature and humidity (temperature between 20.8 °C to 27.0 °C and relative humidity between 25% and 63%). Cleaning of the tested surfaces was not undertaken before sampling to avoid any interference with the accuracy of the results. The incubation time was 72 h at 32.5 °C. The quantitative analyses aimed to determine the colony count of aerobic mesophilic microorganisms, including yeasts and some moulds, expressed as CFU/25 cm^2^.

The second test was conducted in a high school on high-touch surfaces. The microbial burden was examined at normal environmental conditions of temperature and humidity. A One-time sampling was performed on high-touch critical zones, including the table surface and the entrance door handle interior and exterior, just after the last service of the day and before the current cleaning. Cultures were undertaken using a petri dish type PCA and swabbing the indicated surface (25 cm^2^). Incubation time was five days at 30 °C. The quantitative analyses, expressed as CFU/25 cm^2^, aimed to measure the colony count of aerobic mesophilic microorganisms, including fungi.

Results are expressed in percentages of CFU/25 cm^2^ reduction per type of surface using the following formula:

$$\frac{Total\;number\;of\frac{CFU}{{25cm}^{2}}in\;surfaces\;not\;covered\;with\;polyethylene\;adhesive\;film-total\;number\;of\frac{CFU}{{25cm}^{2}}in\;surfaces\;covered\;with\;polyethylene\;adhesive\;film}{\mathrm{total}\mathrm{number}\mathrm{of}\frac{CFU}{{25cm}^{2}}\mathrm{in}\mathrm{surfaces}\mathrm{not}\mathrm{covered}\mathrm{with}\mathrm{polyethylene}\mathrm{adhesive}\mathrm{film}}\times 100$$

### 2.3. Statistical Analysis

Means and standard deviation (SD) were calculated at T0 and T24 to define log reduction, and the Student *t*-test was performed on the three independent experiments conducted for each tested sample treated with microspheres (test) versus untreated test pieces (control) according to the ISO 22196:2011, ISO 20473:2021 and S 90-700 standards. The paired sample *t*-test was undertaken to calculate the significance of the colony count reduction between surfaces treated with polyethylene adhesive film versus none covered surfaces on high-touch areas for in situ results. *p* < 0.005 was considered significant.

## 3. Results

### 3.1. In Vitro Assays

#### 3.1.1. In Vitro Testing of the Antimicrobial Activity of Microspheres-Added Nonporous Surfaces

The tests were conducted on *E. coli* CIP 53.126, *S. aureus* CIP 4.83, resistant strains *S. aureus* ATCC 33591 (MRSA), and *E. coli* (ESBL) under the ISO 22196:2011 (JIS Z 2801:2010) standard conditions at a temperature of 35 ± 1 °C, relative humidity (RH > 90%), contact time of at least 24 h, and inoculum size 10^4^ per cm^2^ and 10^5^ CFU for the tested beauty blender. The results show that the antimicrobial activity of different nonporous surfaces treated with microspheres is significant compared with untreated test pieces, with a log reduction R > 2. The nonporous treated surfaces included polyethylene film, polypropylene plates, latex gloves, and a beauty blender. The diversity of materials used demonstrates the efficiency of this technology applied to vast material types, including medical and pharmaceutical devices, biomaterial coatings, and fabrics used in the health settings, such as personal protective equipment (PPE) (Table 3).

#### 3.1.2. In Vitro Testing of the Antimicrobial Activity of Microsphere-Added Porous Surfaces

The tests were conducted on S. *aureus* CIP 53.156 under ISO 20743 standard conditions at a temperature of 35 ± 1 °C, relative humidity (RH > 90%), contact time of at least 24 h, and an inoculum size of 10^6^ CFU/mL. The results show that the antimicrobial activity (A) of different porous surfaces treated with microspheres is considered significant compared with untreated test pieces, with a log reduction A > 2 for the polycotton tested samples, over-gown polyethylene, and tested gloves, with a > 4 logs reduction. The porous-treated surfaces included nitrile gloves, latex gloves, and polyester and polycotton fabrics (thick and light) (Table 4).

#### 3.1.3. In Vitro Testing of the Antimicrobial Activity of Microsphere-Added Nonporous Surfaces under Real-Life Conditions

The tests were conducted on *E. coli* CIP 53.126 and S. *aureus* CIP 53.156 under the ISO 22196:2011 and NF S90-700:2019 standards by varying the temperature, RH, contact time, and inoculum size. The results show that the increase in the antimicrobial activity of different nonporous surfaces treated with microspheres is time-dependent, with a log reduction R > 2 starting after three hours according to the surface type (Table 5).

#### 3.1.4. In Vitro Simulation of Use (Robustness)

The results of the worst-case scenario simulation tests undertaken under the JIS Z 2801:2010 and ISO 20743 standards indicate that the harsh chemical, physical, and mechanical conditions did not affect the antimicrobial activity of the surface-added microspheres that remained significant with a log reduction >2. (Table 6)

### 3.2. In Situ Assays

#### 3.2.1. In Situ Testing in High-Touch ISO 8 Room Areas

**Figure 2:**
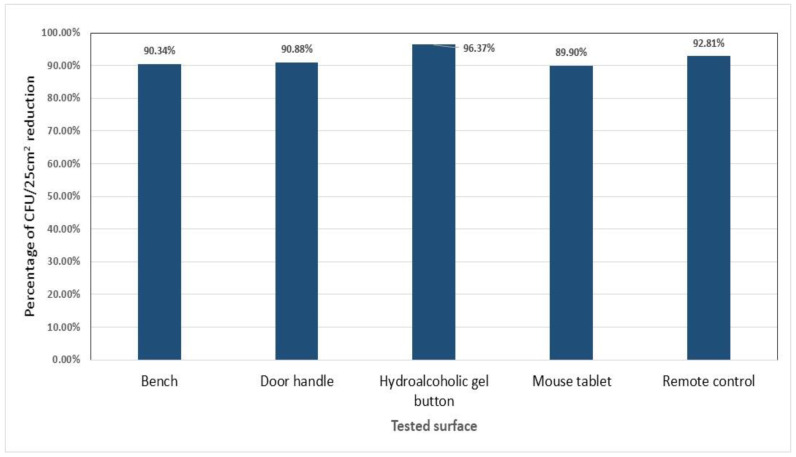
Average percentages of CFU/25 cm^2^ reduction in an ISO 8 room per high-touch surface tested covered with treated polyethylene adhesive film compared with noncovered surfaces.

The level of contamination of noncovered surfaces reached 2–3 logs at baseline. The highest colony count of surfaces without the treated polyethylene film was detected on the remote control (640 CFU/25 cm^2^), followed by the door handle (570 CFU/25 cm^2^), the bench (446 CFU/25 cm^2^), mouse tablet (396 CFU/2 cm^2^), and finally the hydro-alcoholic gel button (331 CFU/25 cm^2^). The results show that the average percentage of CFU/25 cm^2^ reduction in the levels of contamination between the surfaces covered with treated polyethylene adhesive film compared with noncovered surfaces exceeded 90% (*p* = 0.001). The antimicrobial effect was sustainable throughout the duration of the experiment (Figure 2). Details of the tests are shown in Supplementary Table S1.

#### 3.2.2. In Situ Testing in High-Touch Public Zones (High School Self-Service)

**Figure 3:**
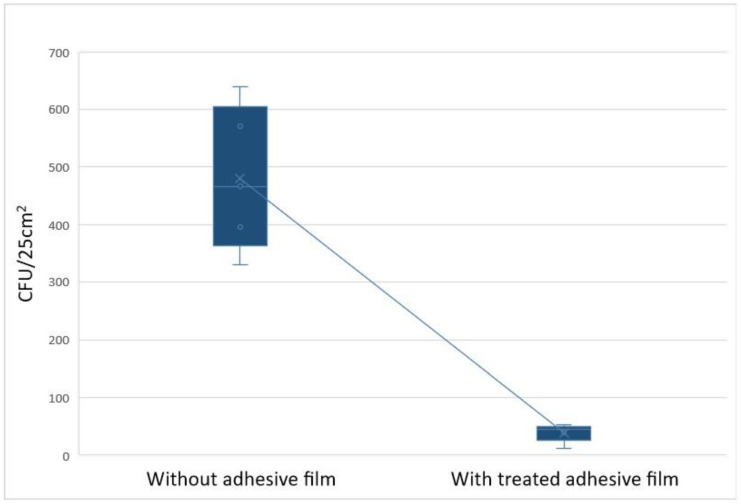
Reduction in CFU/25 cm^2^ in high-use school zones without and with the use of the treated polyethylene adhesive film.

The highest colony count of surfaces without the treated polyethylene film was detected on the interior door handle (>100 CFU/25 cm^2^), followed by the table surface (79 CFU/25 cm^2^) and the exterior door handle (41 CFU/25 cm^2^). The difference in colony counts between surfaces covered with treated polyethylene film compared with noncovered surfaces was 72% (interior door handle), 54% (table surface), and 66% (exterior door handle). These results show that all samples from the treated polyethylene film-added surfaces reached the targeted values set in this experiment of <50 CFU/cm^2^ per tested surface type. A total of 66.7% of noncovered high-touch surfaces had, at baseline, a microbial load higher than 50 CFU/cm^2^ (Figure 3).

## 4. Discussion

The recent COVID-19 pandemic, the spread of antimicrobial resistance [67,68], emerging and re-emerging disease outbreaks [69], and the variable global prevalence of healthcare-associated infections (HAIs) [70,71] mandate a drastic shift in the management of infectious diseases. The microbial contamination of high-touch surfaces in the community [72] and various surfaces and biomedical devices in the healthcare settings, such as catheters, medical instruments, and pharmaceutical outer and inner packages, are potential sources of infection and the leading cause of morbidity, mortality, and healthcare expenditures [71,73]. Microbial persistence in the environment is affected by the conditions of temperature, humidity, initial titer, surface material, and the type of micro-organism, including its ability to form a biofilm [74,75]. These micro-organisms, including resistant strains, can survive for months and sometimes years on dry surfaces [74,75,76]. Studies demonstrated their viability on numerous materials [75], such as fabrics [77,78,79,80], plastics [80,81,82,83,84], steel [85,86,87,88], glass [89], and wood [45]. However, the survival of numerous species on inanimate surfaces remains poorly explored [75]. The efficacy of conventional and automated surface disinfection is influenced by multiple factors [90] and balanced by the exposure of humans to hazards as a result of direct contact with the skin and mucous membranes and airborne inhalation of the chemical residues [1,91] or oxygen-free radicals [92,93]. Recent advances in antimicrobial surfaces are game changers in the management of infectious diseases. However, their efficiency, sustainability and safety need to be demonstrated in vitro and in situ.

This study introduced a patented technology based on oxide mineral microspheres and showed that it could be incorporated into any porous and nonporous surfaces, allowing for a wide array of applicability in different settings and fields, including the pharmaceutical field. The results show the efficiency and sustainability of Pylote-patented oxide mineral microspheres in vitro (1) under three testing standards for porous and nonporous surfaces, including the ISO 22196:2011 (JIS Z 2801), ISO 20743:2021, and NF S90-700:2019 standards (2) under real-life conditions by varying the temperature, humidity, inoculum size and contact time, and (3) simulation of use under harsh chemical, physical, and mechanical testing conditions. The results were aligned in vitro and in situ. The tests were conducted in the community on high-touch surfaces covered with treated polyethylene film versus noncovered surfaces.

### 4.1. In Vitro Assays

#### 4.1.1. In Vitro Efficiency of the Antimicrobial Materials with Added Mineral Oxide Microspheres According to High-Testing Standards

The efficiency of oxide mineral microspheres’ antimicrobial activity was demonstrated according to three quantitative testing standards:

(1) The ISO 22196:2011 (JIS Z 2801) standard evaluates the antimicrobial performance of hard nonporous surfaces and plastics, including gloves, adhesive film (treated Polyethylene film), painted polypropylene plate, varnish solvent film, and a beauty blender;
(2) The ISO 20743:2021 standard applies to textiles, including nonwoven materials, nitrile gloves, over-gown polyethylene, and polycotton tested against the referenced *S. aureus* CIP 4.83;
(3) The NF S90-700:2019 standard tests the bactericidal activity against *S. aureus* CIP 4.83 on nonporous surfaces, such as adhesive film.

The results showed that the oxide mineral microspheres incorporated into numerous materials exhibit significant antimicrobial activity in vitro under standard testing conditions (Log reduction > 2). The efficiency of this technology is related to two main features:

(i) The homogenous dispersion of the ceramic particles in various materials owing to their spherical shape and being initially totally non-agglomerated;
(ii) The close contact between the contaminating micro-organisms, the surface-embedded microspheres, and the microsphere-generated ROS. This feature was proven using a scanning microscope that showed a narrow distance between two microspheres ranging between 0.2 to 1 µm, which is nearly identical to the average size of bacteria (1 µm) [75].

A previously published study demonstrated that the broad spectrum of mineral oxide microspheres has antimicrobial activity against susceptible and resistant Gram-positive bacteria (GPB) and Gram-negative bacteria (GNB), in addition to viruses and fungi [75] in vitro under standard conditions. The tested micro-organisms included GNB, such as *p. aeruginosa* CIP 82·118, *Salmonella enterica* CIP 60·62T, *Branhamella catarrhalis* CIP 73·21T, and *Haemophilus influenza* CIP 102514T, and GPB, such as S. *pyogenes* CIP 56·41T, *S. epidermidis* CIP 68·21, and *Listeria monocytogenes* CIP 82·110T, as well as viruses, including *influenza A virus subtype H1N1* and *Herpes simplex virus 1* (*HSV-1)* and fungi, such *C. albicans* [75].

In order to best evaluate the actual efficiency of the antimicrobial activity, previous studies recommend testing under real-life conditions and preferably setting a new ISO standard for more realistic test outcomes [94,95,96,97].

#### 4.1.2. In Vitro Efficiency of the Antimicrobial Materials with Added Oxide Mineral Microspheres under Real-Life Conditions

The efficiency of porous and nonporous surfaces treated with oxide mineral microspheres was demonstrated in vitro under real-life conditions. When lowering the temperature and humidity and varying the inoculum size, the results showed that the log reduction per cm^2^ increased proportionally to the contact time, where a >2 log reduction was seen starting after 3 h. Multiple studies showed that the conditions determined by the standard assays might not accurately replicate authentic conditions [6,94,97,98,99,100]. However, our results proved the antimicrobial activity of porous and nonporous surfaces with added oxide mineral microspheres under real-life conditions.

Campos (2016) compared the efficacy of antimicrobial thin-film surfaces and highlighted that the activity of these surfaces varies depending on the adapted testing protocol [98]. The effect of temperature and humidity on the antimicrobial activity of silver compared with copper alloy metals by challenging *S. aureus* (MRSA) was assessed under standards and real-life conditions [95]. The results showed that under JIS Z 2801 standard conditions, silver ion-containing materials showed effective antimicrobial activity (log reduction > 5), while no significant response was detected at lower temperatures and humidity levels, which was similar to the indoor environment conditions [95]. Ojeil (2013) showed that tested copper alloy surfaces showed different antimicrobial activity depending on the testing conditions [97]. Michels (2009) demonstrated the high efficacy of antimicrobial materials containing copper alloys, favouring its use in hospital settings under real-life conditions; silver-containing materials that showed high efficacy under standard testing conditions (JIS Z 2801) did not exhibit a significant antimicrobial effect at lower temperatures and humidity [95].

#### 4.1.3. In Vitro Efficiency under Simulation of Use Tests

Published studies tested the activity of antimicrobial surfaces for a duration of more than 10 weeks to replicate the real-life effect of continuous use and demonstrate the sustainability of the antimicrobial effect [99,100]. These tests were perceived as impractical for routine use because they are time- and resource-consuming [6].

In our study, the oxide mineral-added microspheres underwent harsh mechanical, chemical, and physical manipulations that simulate worst-case scenarios (robustness and ageing). According to ASTM F 1980-7, physical ageing at 6 weeks at 50 °C is equivalent to 9 months at 23 °C, and 8 weeks at 50 °C equates to 12 months at 23 °C. The nonporous and porous materials were exposed in vitro to high mechanical ageing through 50 to 100 washes and chemical ageing using Isopropyl alcohol, bleach, and Surfanios Premium^®^ washes. The results demonstrated the robustness and sustainability of the antimicrobial activity over an extended time. Previous assays showed a long-lasting effect of 50 months [58]. The antimicrobial activity was high after exposure to various chemically and mechanically robust conditions. The demonstrated sustainability of the oxide mineral microspheres may be related to the mechanism of action that does not need the release nor consumption of the particles to generate a permanent self-decontaminating surface.

Here, we demonstrated the in vitro efficiency applied to bacteria with acquired resistance, including *E. coli* ESBL and MRSA, and above all, the maintenance of antimicrobial activity when microspheres were added to various porous and nonporous materials. At the same time, we showed a lasting effect according to the assay conditions: high RH and temperature (35 or 36 °C) during the contact time (ISO 22196) and after rapid drying, simulating microdroplet surface contamination and ambient RH and temperature (NF 90-700) for nonporous materials, as well as under ISO 20743 conditions for the porous ones.

### 4.2. Efficiency of the Antimicrobial Materials with Added Oxide Mineral Microspheres In Situ

With the availability of different types of antimicrobial surfaces in the market, manufacturers need to provide evidence of their product’s in situ efficacy for end-user decision-making [54]. A literature search showed scarce studies of the efficiency of antimicrobial surfaces undertaken in situ and primarily in healthcare settings [101]. The in situ experiments targeted high-touch surfaces, considered a reservoir of pathogens, including GNB and GPB-susceptible and resistant strains, viruses, fungi, yeasts, and parasites [102,103,104,105]. These surfaces are commonly disinfected with chemical products that can often provide limited efficacy due to perpetual and instant recontamination [104,105]. The contaminating pathogens may originate from the hands of the personnel in the community and the patient’s microbiota in healthcare settings [72,106]. Micro-organisms may survive cleaning for months and accumulate, providing an additional source of contamination [72].

In this study, in situ experiments were conducted under real-life conditions during working hours. The culture protocol and laboratory testing followed standardized antimicrobial testing to ensure the findings’ validity, comparability, and reliability [54]. In the first in situ experiment (ISO 8 room), the tests were repeated seven times on five different high-touch areas (five with and five without the treated polyethylene film) at specific periods over three months, while in the second (high school self-service), they were carried out once on three high-touch areas (three with and three without the treated polyethylene film).

The results show a significant reduction in the CFU/25 cm^2^ on high-touch surfaces covered with treated polyethylene film compared to the noncovered surfaces. The antimicrobial activity remained sustainable under the testing conditions in situ. These findings from authentic conditions confirm those obtained in the in vitro tests and demonstrate the efficiency of the antimicrobial activity of oxide mineral microspheres under standard and environmental conditions.

## 5. Conclusions

Oxide mineral microspheres are a unique nonrelease, nonleaching, non-nanoparticle-based technology. Porous and nonporous materials with added microspheres showed efficient and sustainable antimicrobial activity in vitro and in situ under high testing standards and real-life conditions. This promising green-tech innovation is incorporated without any change in the manufacturing process regarding any material, offering countless applicability in preventing inanimate surface contamination in the pharmaceutical and medical fields and in different healthcare and community settings. The antimicrobial activity was demonstrated in vitro and in situ against the indicated bacteria according to the testing standards and has also shown effectiveness in a previously published study against susceptible and resistant bacteria, viruses, and to a lesser extent, fungi.

A new testing standard replicating real-life conditions is desirable to show the antimicrobial activity of the microspheres against a broad spectrum of micro-organisms, including additional priority pathogens. The long-term safety of humans and the environment, including the selection of resistant bacteria, is recommended for future research.

## Figures and Tables

**Table 1:** Tested materials and reference materials.

Material	Characteristics	Reference	ISO 22196 Standard			ISO 20743 Standard		Expression of the Results
			Standard Conditions	In Use Conditions	Ageing Conditions	Standard Conditions	Ageing Conditions	
Treated Polypropylene plate	Nonporous	Untreated Polypropylene plate	√	√				/cm^2^
Treated Polyethylene film	Nonporous	Untreated Polyethylene film	√	√	√			/cm^2^
Treated Acrylic coated film	Nonporous	Film Varnish Solvent without adhesive		√				/cm^2^
Treated waterproof	Nonporous	Untreated Polyethylene film	√					/cm^2^
Latex gloves								
Treated Nitrile coated nonwaterproof Gloves	Porous	Untreated Polyethylene film				√	√	/mL
Activity Beauty Blender^®^ (foam)	Porous	Untreated Beauty blender^®^ (foam)	√					/g
Treated Polyurethane Painted plate	Nonporous	Untreated Polyurethane Painted plate		√				/cm^2^
Treated polyester fabric	Porous	Untreated polyester fabric				√	√	/mL
Treated polycotton fabric (light)	Porous	Untreated polycotton fabric				√		/mL
Treated polycotton fabric (thick)	Porous	Standard fabric				√		/mL

**Table 2:** In situ testing experiments.

	In Situ Testing Experiment 1	In Situ Testing Experiment 2
Type of room	Clean room ISO 8	Public area (High school self-service)
Number of employees/customers in the room	2 to 6	More than 100 applicable
Age (years), of employees/customers that access the room	25–50	14–55
Room cleaning routine	Once per month (Specific disinfection with Surfanios Premium)	Daily, after the last service (Current cleaning product)
Room cleaning during the testing period	Cleaning of the tested surfaces was not undertaken prior to sampling to avoid any interference and inaccuracy in the results. Sampling between 8 h and 9 h AM Maintain the cleaning disinfection process with Surfanios premium (monthly)	Cleaning and disinfection of the tested surfaces were not undertaken prior to sampling to avoid any interference and inaccuracy in the results.
Tested surfaces	Five surfaces covered with Treated Polyethylene film were tested, including the bench in the changing room, door handle, hydroalcoholic gel button, mouse tablet, remote control	Three surfaces covered with treated Polyethylene film were tested, including interior door handle, exterior door handle, table
Reference	Corresponding surfaces without treated polyethylene film	Corresponding surfaces without treated polyethylene film
Total number of cultures	35 (5 per week–7 weeks) treated vs. 35 non treated	3 treated vs. 3 nontreated
Culture protocol	Cultures were carried out between 8:00 and 9:00 a.m. during normal working hours every Monday or Tuesday from April to June 2021	Cultures were carried out once (11 June 2020) at the end of the last shift
Testing conditions	The temperature and humidity were recorded. Depending on the season, the temperature varied between 20.8 °C to 27 °C and the humidity between 25% and 63%	Not recorded

**Table 3:** Log CFU after 0 h (control C0) and 24 h of contact with different types of nonporous surfaces (control C24 and assay A24) and log reductions per cm^2^.

ISO 22196:2011						
Tested Samples	Bacterial Strains	C0	C24	A24	R	*p*
Treated Latex gloves	*E. coli* CIP 53.126	3.82 ± 0.03	5.97 ± 0.029	0.00 ± 0	5.97 ± 0.029	<0.001
Treated Polyethylene film	*E. coli* CIP 53.126	4.06 ± 0.011	5.87 ± 0.08	0.00 ± 0	5.87 ± 0.08	<0.001
Treated Beauty blender (foam)	*E. coli* CIP 53.126	4.27 ± 0.02	6.48 ± 0.07	<0.62 ± 0.02	>5.86 ± 0.09	<0.001
Treated Beauty blender (foam)	*S. aureus* CIP 4.83	4.52 ± 0.09	4.61 ± 0.018	<0.60 ± 0.017	>4.01 ± 0.020	0.001
Treated Polyethylene film	*S. aureus* CIP 4.83	3.88 ± 0.03	4.65 ± 0.18	1.26 ± 0.64	3.39 ± 0.61	0.011
Treated Polypropylene plate	*S. aureus* CIP 4.83	3.80 ± 0.07	3.47 ± 0.029	<0.58 ± 0.55	>2.89 ± 0.26	0.003
Treated Polypropylene plate	*S. aureus* ATCC 33,591 (MRSA)	5.26 ± 0.01	4.21 ± 0.30	<1 ± 0.00	>3.01 ± 0.30	0.003
Treated Polypropylene plate	*E. coli* (ESBL)	5.03 ± 0.02	6.96 ± 0.04	3.13 ± 0.47	3.83 ± 0.44	0.004

MRSA: Methicillin-resistant *Staphylococcus aureus*; ESBL: extended spectrum beta-lactamase. Log reduction = R = C24 − A24; Antibacterial activity is significant if R > 2. Results are expressed as means (SD) of three independent experiments (*n* = 3).

**Table 4:** Log CFU after 0 h (control C0) and 24 h (control C24 and assay A24) of contact with different types of porous surfaces and log reductions per cm^2^ for tests with *S. aureus* CIP 4.83.

ISO 20743:2021						
Tested Samples	C0	C24	T0	T24	A	*p*
Treated Nitril gloves	4.71 ± 0.06	6.43 ± 0.21	4.29 ± 0.29	>1.40 ± 0.17	>4.61 ± 0.66	0.007
Treated Latex gloves	4.99 ± 0.20	6.36 ± 0.32	4.35 ± 0.21	1.67 ± 0.49	4.05 ± 0.85	0.014
Treated polyester fabric	4.99 ± 0.10	6.07 ± 0.30	4.13 ± 0.16	2.08 ± 0.72	3.18 ± 0.83	0.023
Treated polycotton fabric (light)	4.99 ± 0.10	6.07 ± 0.30	4.03 ± 0.07	2.42 ± 0.57	2.73 ± 0.79	0.028
Treated polycotton fabric (thick)	4.99 ± 0.10	6.07 ± 0.30	4.14 ± 0.22	2.56 ± 0.17	2.77 ± 0.25	0.003

Log reduction = A = (logC_24_ − logC_0_) − (logT_24_ − logT_0_). Efficacy of antibacterial properties (A): Significant: 2 ≤ A ≤ 3; High level: A ≥ 3. Results are expressed as means (SD) of three independent experiments (*n* = 3) for *S. aureus* CIP 4.83.

**Table 5:** Log reductions per cm^2^ after varying the testing conditions (temperature, RH, contact time, and inoculum size) for different types of nonporous surfaces.

ISO 22196:2011							
	Bacterial Strain	Temperature	RH	Inoculum Concentration	Contact Time	Log Reduction	*p*
Treated Acrylic coated film *	*E. coli* CIP 53.126	36 ± 1 °C	24%	2.6 × 10^2^ CFU/cm^2^	1 h	0.47 ± 0.17	0.042
Treated Acrylic coated film *	*E. coli* CIP 53.126	22 ± 2 °C	46%	10⁴/cm^2^	24 h	3.95 ± 0.61	0.008
Treated Polyurethane Painted plate	*E. coli* CIP 53.126	36 ± 1 °C	>90%	1 × 10^2^ UFC/cm^2^	1 h	0.86	
					2 h	1.33	
					3 h	3.35	
Treated Acrylic coated film	*E. coli* CIP 53.126	36 ± 1 °C	>90%	1.3 × 10^2^ CFU/cm^2^	30 mn	0.14	
					2 h	0.4	
					6 h	2.22	
Treated Polypropylene plate *	*E. coli* (ESBL)	36 ± 1 °C	>80%	10^5^/cm^2^	4 h	1.83	0.001
Treated Polypropylene plate *	*S. aureus* ATCC 33,591 (MRSA)	36 ± 1 °C	>80%	10^5^/cm^2^	4 h	0.57	<0.001
**NF S90-700:2019**							
Treated Acrylic coated film *	*S. aureus* CIP 4.83	20 ± 2.5 °C	50%	10^5^/cm^2^	24 h	2.16	0.013

* Results of three independent experiments.

**Table 6:** Worst-case scenarios in vitro simulation test. Log CFU numerations after 0 h (control C0) and 24 h (control C24 and assay A24) of contact with different types of surfaces and log reductions per cm^2^ with *E. coli* CIP 53.126 (nonporous surfaces) and *S. aureus* CIP 4.83 (porous surfaces).

**ISO 22196:2011**								
**Nonporous Surfaces**								
	**Simulation of Use**	**Bacterial Strain**	**C0**	**C24**	**A24**	**R**		***p***
Treated Acrylic coated film	High mechanical ageing	*E. coli* CIP 53.126	3.98 ± 0.03	5.87 ± 0.05	0.00 ± 0	5.87 ± 0.05		<0.001
Treated Polyethylene film +	12 weeks at 50 °C	*E. coli* CIP 53.126	4.07 ± 0.03	5.70 ± 0.03	0.43 ± 0.44	5.27 ± 0.47		0.003
Treated Polyethylene film ++	12 weeks at 50 °C	*E. coli* CIP 53.126	4.07 ± 0.03	5.70 ± 0.03	1.35 ± 1.87	4.35 ± 1.88		0.057
Treated Polyethylene film	Isopropyl alcohol treatments *	*E. coli* CIP 53.126	4.05 ± 0.01	5.87 ± 0.08	0.00 ± 0	5.86 ± 0.08		<0.001
	Bleach treatments *	*E. coli* CIP 53.126	4.05 ± 0.01	5.87 ± 0.08	0.00 ± 0	5.86 ± 0.08		<0.001
	Surfanios Premium^®^ treatments *	*E. coli* CIP 53.126	4.05 ± 0.01	5.87 ± 0.08	0.00 ± 0	5.86 ± 0.08		<0.001
**ISO 20743:2021**								
**Porous Surfaces**								
	**Simulation of Use**		**C0**	**C24**	**T0**	**T24**	**A**	***p***
Treated Latex gloves	After wash at 40 °C and dry at room T°	*S. aureus* CIP 4.83	4.99 ± 0.20	6.36 ± 0.32	4.29 ± 0.15	1.43 ± 0.23	4.22 ± 0.11	<0.001
Treated polyester fabric	After 50 treatments	*S. aureus* CIP 4.83	4.09 ± 0.16	5.45 ± 0.22	3.45 ± 0.34	>1.30 ± 0.00	>3.51 ± 0.05	<0.001

T°: temperature; + without adhesive and without anti-UV; ++ without adhesive and with anti-UV; * 100 times. Twelve weeks at 50 °C = 18 months 23 °C; six weeks at 50 °C = 9 months at 23 °C; eight weeks at 50 °C = 12 months at 23 °C.

## Data Availability

Data is contained within the article or supplementary material.

## Supplementary Materials

The following supporting information can be downloaded at: https://www.mdpi.com/article/10.3390/pharmaceutics15041261/s1, Table S1: Tested high touch zones under authentic environmental conditions.
