# Supplementary material for: Demonstrating the In Vitro and In Situ Antimicrobial Activity of Oxide Mineral Microspheres: An Innovative Technology to Be Incorporated into Porous and Nonporous Materials
Source: Pharmaceutics. 2023 Apr 17;15(4):1261. doi: 10.3390/pharmaceutics15041261 (PMC10144421; doi:10.3390/pharmaceutics15041261)
Supplement: Supplementary file 1 [file pharmaceutics-15-01261-s001.zip › pharmaceutics-2277482-supplementary.pdf]

pharmaceutics

MDPI

Supplementary Materials: **Demonstrating the In Vitro and In Situ Antimicrobial Activity of Oxide Mineral Microspheres: An Innovative Technology to Be Incorporated into Porous and Nonporous Materials**

Katia Iskandar, Sophie Pecastaings, Céline LeGac, Sylvie Salvatico, Catherine Feuillolay, Mylène Guittard, Loïc Marchin, Marc Verelst, and Christine Roques

Table S1. Tested high touch zones under authentic environmental conditions

	**Tested high touch zones**									
	**Bench**		**Door handle**		**Hydroalcoholic gel button**		**Mouse Tablet**		**Remote control**	
**Conditions of temperature and humidity**	**CFU/25cm²**									
To: 20.8°C - 24°C RH: 25% - 31%	56	7	54	**1**	0	0	300	8	3	40
To: 21°C - 25°C RH:25% - 36%	35	16	3	6	23	3	14	20	11	0
To: 21°C - 25°C RH:26% - 34%	28	1	60	1	7	0	4	6	3	0
To: 22 °C - 26.5 °C RH:32% - 50%	7	5	>300	13	1	0	13	1	12	0
To: 20.5 °C - 25°C RH: 34% - 46%	31	11	109	5	1	5	2	3	0	9
To: 21°C - 26°C RH:31% - 41%	3	2	79	0	6	2	15	1	15	0
To: 23°C - 27 °C RH:43% - 63 %	9	4	7	33	1	1	0	1	9	0
**Total number of CFU/25cm²***	**466**	**45**	**570**	**52**	**331**	**12**	**396**	**40**	**640**	**46**
**% of microbial reduction**	**90.34%**		**90.88%**		**96.37%**		**89.90%**		**92.81%**	

Columns in gray are tested zones without polyethylene adhesive film. Column in white are tested zones with polyethylene adhesive film fim;The average percentage of CFU/25cm² reduction is 92.06%, * p=0.001.
